# Supplementary figures and images for: Dementia risk and dynamic response to exercise: A non-randomized clinical trial
Source: PLoS One. 2022 Jul 8;17(7):e0265860. doi: 10.1371/journal.pone.0265860 (PMC9269742; doi:10.1371/journal.pone.0265860)

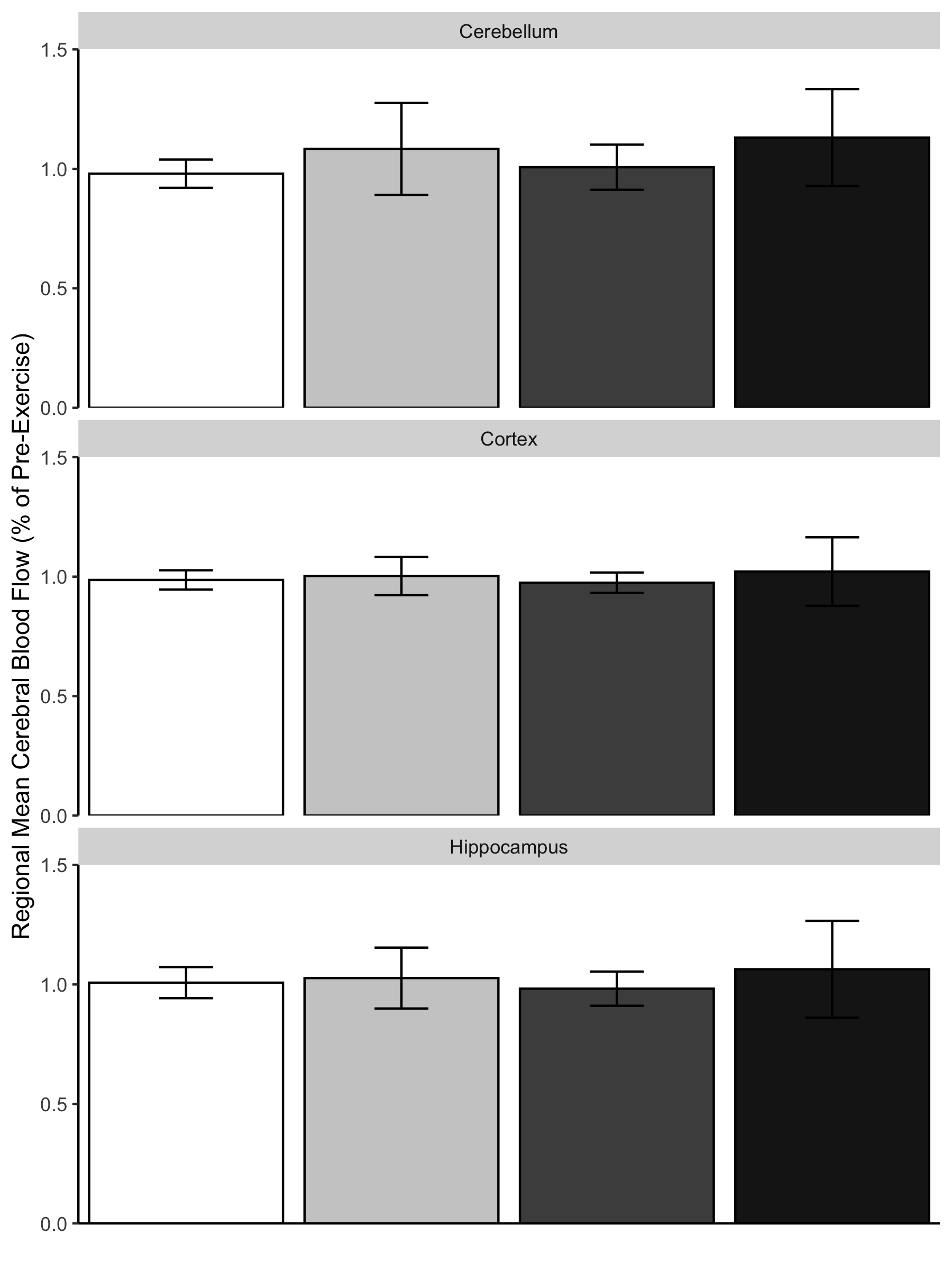

Supplement: S1 Fig — The figure shows relative cerebral blood flow in three regions of interest. Pre- and post-exercise time frames are equivalent, ~12 minutes of arterial spin labeling data collection. The hippocampus demonstrated an increase in post-exercise cerebral blood flow over pre-exercise in APOE4 carriers only (p = 0.05). The white bar is pre-exercise for APOE4 non-carriers. The light gray bar is post-exercise for APOE4 non-carriers. The dark graybar is pre-exercise for APOE4 carriers. The black bar is post-exercise for APOE4 carriers. Error bars are standard deviation. Cerebral blood flow is shown in percentage of the second PCASL acquisition before exercise. (TIF) [file pone.0265860.s002.tif]
