# Supplementary material for: Dementia risk and dynamic response to exercise: A non-randomized clinical trial
Source: PLoS One. 2022 Jul 8;17(7):e0265860. doi: 10.1371/journal.pone.0265860 (PMC9269742; doi:10.1371/journal.pone.0265860)
Supplement: S1 Protocol — (PDF) [file pone.0265860.s003.pdf]

STUDY PROTOCOL – Dynamic Arterial Measurement in Cerebrum (DYNAMIC)

PI: Eric Vidoni, PT, PhD
Co-I: Sandra Billinger, PT, PhD
Laura Martin, PhD
Rebecca Lepping, PhD
Phil Lee, PhD
Jill Morris, PhD

Version: 20200921

Significance

Scope of the Problem

The number of Americans 65 years and older will double in size over the next 40 years.[1] Aging brings increased cognitive decline.[2] Alzheimer's disease (AD), the most common type of dementia, is of particular concern to the health care system, with an expected two-fold increase in prevalence over the next 30 years and high direct and indirect costs of care.[3,4] We must find effective interventions to reduce the burden of AD on our society and economy. Much like the field of heart disease 30 years ago, reducing AD prevalence through primary prevention is now the focus of broad research investment. There is evidence that risk of cognitive decline can be moderated by lifestyle interventions such as physical exercise,[5-12] although the extent literature is not conclusive.[13-16] Conflicting evidence is likely a result of imprecisely controlled interventions and a lack of understanding about key target mechanisms. There are several potential mechanisms that may relate exercise with brain health. Our aims are designed to specifically explore cerebral blood flow (CBF) as a potential mechanism of exercise effects on brain and cognition, and is specifically related to the APOE allele, the strongest genetic risk factor for late-onset AD.. Understanding how exercise promotes cognition and brain health is essential to fully capitalize on its benefits for the treatment and prevention of AD.

Exercise Represents a Promising Strategy for Reducing Risk of Cognitive Decline

Exercise --defined as planned, repetitive physical activity-- has a biologically plausible and temporal relationship with heart disease,[17] atherosclerosis,[18] stroke,[19] diabetes,[20,21] obesity,[22,23] hypertension,[24] depression,[25] all established risk factors for dementia.[4] Aerobic exercise is among the most important and cost effective tools available for chronic disease management.[26] Our work along with others' strongly suggests that moderate-intensity aerobic exercise benefits brain health and cognition.[10,11,27-30]

Exercise, Cognition and Brain Health: Longitudinal studies suggest physical activity is positively associated with cognition and brain health and reduced risk of cognitive decline and dementia.[5,6,31-38] Perhaps more compelling, RCTs have repeatedly demonstrated benefits of aerobic exercise on cognition (particularly in executive and visuospatial function) and measures of brain health such as whole brain and hippocampal volume in people with and without cognitive decline.[7-11,13,39-43]

Cerebrovascular Health is Vital for Cognitive Function and Brain Health

The brain is intolerant of ischemia and highly dependent on healthy blood supply, requiring up to 25% of whole body resting O2 consumption.[44] Consequently, CBF is highly regulated to protect against hypoperfusion.[45,46] There is increasing evidence that cerebrovascular dysfunction contributes to AD. In animal models, β-amyloid disrupts cerebrovascular regulation.[47-49] In humans, morphological changes occur in cerebral arteries in AD.[50,51] Additionally, CBF is lower in people with neurodegenerative diseases such as AD, and cerebrovascular pathology precedes and accompanies neurodegeneration.[52-58] Reasons for lower cerebral blood flow are unclear but may include diminished heart function,[59] atherosclerosis risk,[60,61] and deficits in vessel wall health.[62,63]

Our previous work suggests the presence of β-amyloid, in otherwise cognitively intact individuals, is associated with altered cerebrovascular regulation measured at rest and during exercise (see Figure 1).[64] Further investigation of CBF regulation is therefore vital to understanding cognitive decline and the development of AD.

Aerobic exercise effects on CBF may only become apparent when dynamically measured during exertion.[65] Measuring CBF following exercise rather than at rest may provide a more sensitive and relevant measure for detecting differences in subjects at risk for AD.

<u>*Cerebral Blood Flow Improves with Exercise:*</u> There is considerable interest in precisely defining cerebrovascular adaptations to aerobic exercise and how those adaptations may support cognitive function.[66,67] We know that sedentary older adults demonstrate decreasing CBF over time.[54,68-70] Habitual exercise appears to increase CBF,[68,70,71] and older athletes who take time off of training quickly experience reductions in CBF.[72] Additionally, heart disease and stroke patients improve CBF while completing their cardiac rehab program.[73,74] However, evidence from prior aerobic exercise intervention trials with older adults have been mixed, some reporting increased CBF at rest,[75] and others reporting no difference.[76]

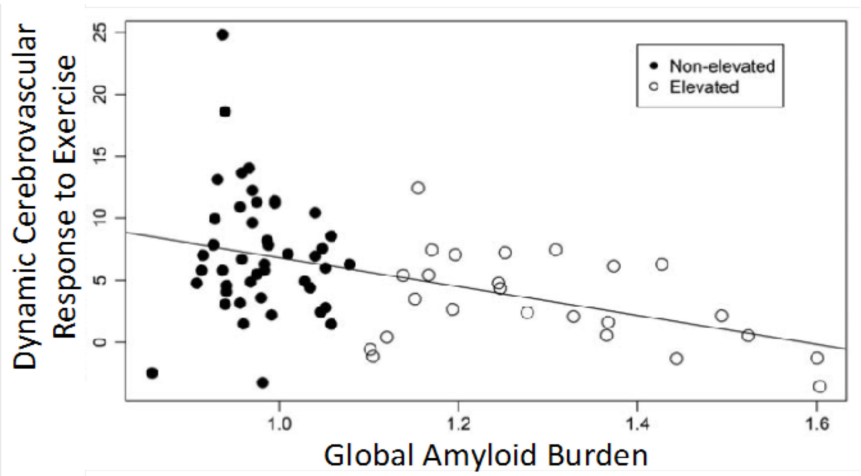

Figure 1. CBF response to exercise (cm/s, the ordinate) is lower in cognitively normal individuals with greater β-amyloid burden (standard uptake value ratio, the abscissa). Open circles are individuals with elevated β-amyloid. Sisante et al. J Cereb Blood Flow Metabol 2017.

Our work demonstrates the importance of measuring dynamic response to exercise. Drs. Billinger and Vidoni recently described the dynamic change in a measure of CBF with onset of exercise in young and older individuals (Figure 2)[46] demonstrating that older adults have a pronounced blunting of CBF during exercise that was less evident at rest.

Increased CBF can be measured by arterial spin labeled (ASL) MRI. Optimal imaging parameters to capture CBF response to acute exercise using ASL, identifying a 20% increase immediately following an acute bout of exercise. [77]

Drs. Martin and Lepping on our team have conducted a pilot study (n=11) to examine changes in blood flow following acute exercise (50-70% max HR) compared to quiet rest in smokers. ASL was performed ~30 minutes after exercise. Analyses revealed a pattern of increased CBF generally, and in ACC and precentral gyrus specifically, p <0.16.

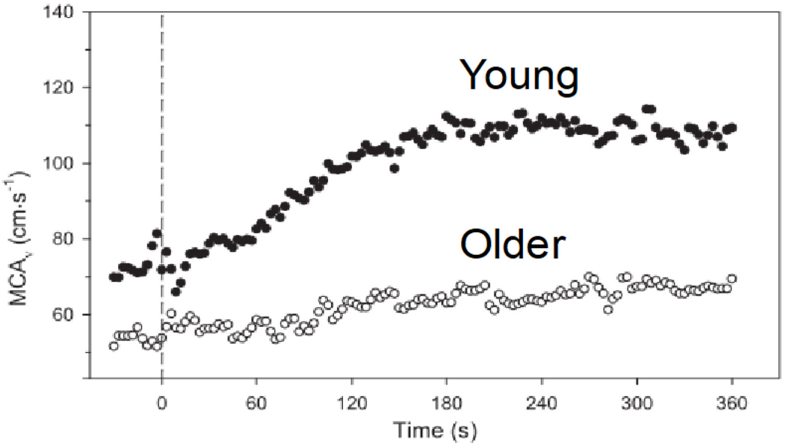

Figure 2. Middle cerebral artery velocity response at the onset of exercise (cm/s, the ordinate) is diminished in older individuals. Dashed line is onset of moderate exercise. (Billinger et al. J Appl Physiol 2017.)

This collective body of work demonstrates our ability to capture CBF change with highly coordinated, precisely timed acute exercise protocols. It also provides a compelling case for exploring the cerebrovascular exercise response in relationship to AD risk.

<u>*Interaction of CBF, neurotrophins, and APOE Genotype:*</u> The **scientific premise** underlying this proposal is that CBF and blood-based biomarkers such as VEGF, BDNF, and IGF1 are interrelated mechanisms driving chronic aerobic exercise effects on brain health and cognition. Several lines of evidence are now converging to point to these interacting factors as critical for supporting cognition and brain health into old age.

There are well-documented differences in cerebral blood flow (CBF) and tissue oxygenation based on age or APOE genotype,[54,78-80] with deleterious consequences for cognition.[54,81] Cerebrovascular endothelial cells respond to the shear force stress on the vessel walls by releasing BDNF.[82,83] Further, carotid occlusion extinguishes exercise-induced BDNF increases in the brain, strongly suggesting that increased cerebral blood flow is a key trigger of BDNF release.[83] Therefore, maintenance or improvement in CBF is likely an important mediator brain health and cognitive effects as we age.[75,84,85]

Because VEGF is a salient promoter of vascularization, it is an evident candidate for association with CBF. Previous work demonstrates that rodent neuro- and angiogenesis are coupled with CBF following an exercise

intervention.[85,86] However, it is unclear if APOE4 moderates this relationship. Recently it has been suggested that APOE4-driven pathologies are mediated by a VEGF-dependent pathway but more work is needed [87,88]

It is known that the ApoE protein is important for maintaining cerebrovascular integrity.[63] Additionally, different isoforms of the ApoE protein appear to regulate expression and release of BDNF, at least in vitro.[89] And, though we know individuals with cognitive impairment (MCI or AD) have lower serum BDNF levels[90-92] and tissue pro-BDNF,[89] large studies have failed to demonstrate a relationship between E4 carriage and circulating BDNF levels in cognitively normal individuals.[93,94]

Approach

We will significantly reduce participant burden and speed execution by heavily leveraging the infrastructure of the KU ADC, one of 31 NIH-designated Alzheimer's Centers of Excellence. The KU ADC follows over 400 individuals with annual evaluations as part of our Clinical Cohort. We have extensive experience in neuroimaging and exercise testing.[11,34,95-97] As presented previously, this work is a natural extension of our robust research program and will enhance our ability to measure acute exercise response. We are confident in our timing and are currently conducting a study that requires blood draws before and 15min after exercise.

Overall Study Design: Up 90 participants will be recruited from the KU Alzheimer's Disease Center (KU ADC) and the community. For this study, participants will attend one visit at the Hoglund Brain Imaging Center on the University of Kansas Medical Center campus. Performing all activities at the single visit reduces burden on participants and allows capture of key biomarkers in temporal relationship following a rigorous, time-sensitive protocol. Inclusion and exclusion criteria are further detailed in the Human Subjects section.

Recruitment: E4 carriers make up approximately 33% of the Clinical Cohort. We have an estimated 90 non-demented individuals that we can approach with an E4 allele. Based on our experience, ~60% of eligible Cohort enrollees will consent to the proposed project. Additionally, Dr. Vidoni directs a robust Outreach & Recruitment Core which maintains a list of 7500+ individuals who have expressed interest in dementia research.[98] We project enrollment of 1-2 individuals per week, completing by mid-Year 2.

Genotype will not be disclosed to the participant. Study staff with direct participant contact will remain blinded to genotype. Because APOE4 does not have equal penetrance in our Cohort, we will begin by approaching an E4-enriched contact list created by the KU ADC Statistics Core (2 E4 carriers:1 non-carrier). A co-investigator not in contact with PHI (Morris) will monitor enrollment for E4 carriage and request contact list adjustments as necessary to achieve haplotype balance. We will exclude individuals with an E2 allele. We will make efforts to preferentially match APOE4 genotype groups based on sex.

In some instances, potential participants who have not been characterized in the past 6 months or at all by the KU ADC through study #11132 may express interest in participating. In these instances, the participant will be invited to identify a study partner so that the PI can adjudicate cognitive performance, the QDRS[99]. A study partner who knows the participant well will be required to answer the QDRS questions over the phone.

We will use an approved phone screening consent from the potential participant to get basic demographic and screening information and the name of a study partner who knows the potential participant well. We will then use an approved phone consent for the study partner and ask questions regarding the cognition and function of the potential participant (approximately 10 minutes) to qualify the individual for the study. Given that all participants will have already been initially prescreened through our Recruitment and Eligibility Database (#140406) we do not believe we will encounter any individuals who cannot consent for themselves.

Study Protocol

After passing initial screening participants will attend a single study visit. Written informed consent will be provided either during the visit, or prior to the visit via the telephone or an internet meeting (e.g. Zoom). If consent is performed prior to the visit, the study team will either send a paper copy of the consent to the potential participant or provide a REDCap link to a REDCap for with the consent downloadable one week before the visit. If the participant chooses to have a paper copy mailed, the staff member will perform consenting as usual over the phone or internet and the participant will return the consent to the staff before or at the visit. If the potential participant chooses to receive an email link, the staff member will perform

consenting as usual over the phone and the participant will electronically sign the REDCap form using a signature box.

Strict timing of the protocol is necessary to capture response to acute exercise for CBF [ASL] (Table 1). ASL measures are time-sensitive and have been planned based on prior work.

Table 1. Steps in the Protocol are Highly Time-dependent (Exercise warm-up start begins timing [T0])	Time
1. Staff conduct informed consent and MRI safety screen. Then the participant changes into approved MRI compliant clothing.	Variable
2. Cognitive tests are administered on an iPad. Some participants who have not had a recent Clinical Cohort evaluation may be required to complete additional paper based cognitive assessments (~20 minutes) to index cognitive function.	Variable
3. The participant is escorted to the MRI suite, situated on the imaging table and a baseline scanning is performed for about 20 minutes.	Variable
4. Vitals are taken. A catheter is placed in the antecubital or other appropriate vein for ~10mL blood draw (if participant declines or is unable, repeated sticks may be used). An additional ~6mL blood draw will be taken for those not previously genotyped.	Variable
5. The participant moves to the space with exercise device. Seat height is adjusted, and participant is familiarized with device and cadence.	
6. The participant works at a certain cadence and resistance according to an algorithm for a 2 min warm-up.[64] Then, staff increases workload incrementally over 3 min to reach 65-75% of age predicted heart rate maximum (APHRmax).	T0-T5
7. The participant maintains cadence while study staff titrate workload to maintain 65-75% APHRmax for 15 min. After 15 min, ~10mL blood and vitals are taken immediately. The participant cools-down self-selected cadence and low load for 3 min.	T5-T28
8. The participant returns to the MRI table. We have allotted 8 minutes for repositioning understanding that this population may need additional time to maximize comfort.	T28-T36
9. At time T36, additional MRI sequences in succession are acquired. Scan time is approximately 24 minutes	T36-T60
10. At time T60, participants return to the testing room for cognitive testing and ~10mL blood draw and vitals.	Variable

Exercise Dose: Several different aerobic workloads (from ~50%[100] to 100% of maximum capacity[101,102] and durations (15 minutes[103,104] to 4 hours [105]) have been used in prior studies of response to acute exercise. Our experience informs the selected exercise parameters as achievable by older adults.[34,46,96,106]

Participants will exercise for 15 minutes at 65-75% of age-predicted heart rate maximum (APHRmax) calculated as 208-0.7*Age or 220 minus age to be determined by tester based on medications, age, and perceived fitness level.[107] We will use either an electronically braked cycle ergometer (Lode, Groningen, Nederland) or a NuStep recumbent stepper (NuStep, Ann Arbor, MI) to maintain precise control over workload and heart rate.[77] We will also a small amount of water as needed after exercise to account for any dehydration that might affect blood volume measured by MRI.

Blood Draw: Following catheter placement prior to exercise, blood (~10mL) will be drawn into a serum-separating vacutainer tube. An identical blood draw will be performed immediately after exercise and after post-exercise cognitive testing. Serum will be processed and frozen at -80C until further analysis using colorimetric ELISA for our identified blood-based biomarkers. Some blood will be banked for future analysis.

Blood draws will be performed at Hoglund by appropriately trained staff. If the participant has not previously been genotyped as part of a KU ADC study, an additional ~6mL of blood will be drawn at baseline for the purposes of genotyping.

Imaging Parameters: The investigative team has extensive experience with imaging and preliminary evidence to demonstrate the necessary skill set for capturing time-dependent ASL changes due to exercise. We will employ imaging sequences most suited to capture anatomical and brain blood flow, lasting ~20 minutes before exercise and ~30 minutes after exercise.

Cognitive Testing: Evidence suggests that subtle, transient cognitive effects of exercise are detectable immediately following an acute exercise bout in older adults.[108-110] We will employ a short battery of cognitive tests to capture exercise-related change in: executive control, episodic memory, and processing speed. The battery will last ~20 minutes, depending on participant speed.

Our primary cognitive measure will be the response time cost of incongruent stimuli on the Flanker Test. The Flanker Test is an executive function task that measures inhibitory control [111,112] with a well-documented history of indexing change following exercise intervention[40,112-117] (our long term goal), and early in AD.[118,119]

Aim 1: Characterize CBF change after exercise. Our primary outcome of interest for Aim 1 is global CBF percent change from baseline from rest to immediately after exercise. Secondary outcomes will be absolute change in CBF (ml/100g tissue/min) and spatial coefficient of variation (an indirect measure of arterial transit time).[74] We hypothesize that older participants have blunted exercise-induced change in CBF.

Aim 1 Statistical Analyses: Custom software utilizing the ASL Toolbox for SPM 12 will quantify global CBF.[120] Our primary and secondary outcome measures are continuous, and thus we will use linear mixed models (LMM). We will include a random intercept coefficient to allow for each pre-post exercise pair to account for individual baseline differences. Age group will be modeled as a fixed effect. Other analysis techniques may be employed as they become available.

Sample Size: To our knowledge, there are currently no peer-reviewed reports of genotype-based CBF differences in response to acute exercise. However, perfusion measures in genetic risk for AD (APOE e4) have been performed previously and can form the basis of a reasonable power analyses. Thus, our sample size was determined based primarily on feasibility and preliminary data. First, KU is uniquely suited to perform this study. We have had success in recruiting and characterizing individuals for imaging and exercise studies under complex protocols. Second, two prior cross-sectional estimates of the relationship of perfusion and e4 carriage have delivered similar effect sizes (d=1.0).[78,79] Given this power, we expect to be able to discern differences between sex, and between older adults with and without cognitive impairment.

Exploratory Aim: Describe the relationship between CBF and cognitive performance after acute exercise. Our primary outcome will be the response time on the Eriksen Flanker Task. We will explore these cognitive effects in individuals, assessing how CBF and our blood-based biomarkers relate to cognitive improvements. These data will set the stage for future work assessing who responds to habitual exercise with cognitive improvements, and through what mechanisms.

Exploratory Aim Statistical Analyses: We will use the same least squares regression approach as in Aim 2.

Data Management: Study data will be collected and managed using the Research Electronic Data Capture (REDCap) web-based, electronic data capture tools.[121] Imaging data will be archived and backed up on a dedicated XNAT server operated by the Hoglund Brain Imaging Center. Hoglund also maintains an imaging analysis server that provides researchers access to up-to-date imaging software (e.g. SPM). The PI has over 10 years of experience with REDCap, XNAT, and imaging analysis. Both departments ensure data security by managing all data HIPAA compliant servers that have role-based access and regular backup.

Participant Compensation

Participants will be compensated $100 after the study visit through the Greenphire Clin Card system.

Protection of Human Participants

a. Human Participants Involvement and Characteristics:

For this study, we plan to enroll up to 80 participants from our screening population (the KU ADC Clinical Cohort, and Recruitment Database), other research databases on the KUMC campus (e.g. Pioneers or FitHawks with permission) and the community. Goal enrollment is:

1) up to 20 individuals, age 18-65
3) 60, age 65-85 (approximately equal APOE4 carrier and APOE4 non-carrier)

Inclusion Criteria:

- Age 18-85 (inclusive)
- English speaking
- Normal or corrected hearing or vision,

Exclusion Criteria:

- Cognitive impairment (adjudication can include CDR>0 or QDRS equivalent in last 6 months) unless under age 50 for which cognitive adjudication by above means is not necessary).
- Clinically significant cognitive or psychiatric illness, or other neurological disorders that have the potential to impair cognition (e.g., Parkinson's disease, stroke defined as a clinical episode with neuroimaging evidence in an appropriate area to explain the symptoms).
- Myocardial infarction or symptoms of coronary artery disease (e.g. angina) in the last 2 years.
- ACSM Cardiac Risk stratification of High Risk, unless confirmation of doctor that moderate exercise is appropriate.
- Significant pain, metabolic, or musculoskeletal disorder that would contraindicate exercise.
- MRI exclusions or claustrophobia
- Any clinician prescribed activity restrictions unless clearance is obtained for study
- Anti-coagulant use

b. Sources of Materials:

<u>Description of research materials</u>: Magnetic resonance images, blood/genotype, and cognitive performance will be the only material collected for analyses. Health information such as health/medical history, medication, and medical records will be collected with consent. Participant data will be coded, and only the study team will have access to the codes. All data will be collected for this research study under conditions approved by the KUMC IRB.

<u>Management, analysis and protection of materials, including data management plan:</u> The study will be submitted for review to the KUMC IRB, and will be conducted in accordance with Good Clinical Practice and HIPPA guidelines. Participants will provide written informed consent prior to entering the study. All identifiable files, data, and tissue will be coded and stored in secure locations. The PI will be responsible for the quality of the data and will supervise the data acquisition with the help of the study team. The REDCap database system uses data validation and review algorithms to support data quality. There is no billing of participants, their families, or third party payers for the research assessments.

c. Potential risks:

Moderate-Intensity Exercise: Potential risks of the exercise stimulus as well as the exercise intervention include unpredictable changes in blood pressure or heart rhythm, myocardial infarction and death as well as the less serious problems of fatigue, muscle soreness, tripping, falling, and other injury to tendons, ligaments, joints, and muscles. This protocol requires effort well below maximal and is within standard exercise prescription for healthy adults. Risk is minimized by our Exclusion Criteria which preclude enrollment of individuals at risk for cardiac or orthopedic complications. There is no statistical information regarding serious complications during exercise testing in patients without ischemic coronary heart disease. A review of the experience of 30 cardiac rehabilitation programs reported information on 13,750 participants who accumulated 1,629,634 patient hours of supervised exercise. A total of 50 cardiac arrests were observed, 42 of which were successfully resuscitated while 8 were fatal. Events are extremely rare, and participants will be screened for risk of cardiovascular disease and symptoms. Emergency equipment, including defibrillator, and ambulance plan are available for all participants.

Neuroimaging: MRI scanning is not generally associated with any health risks. Trained technicians at the Hoglund Brain Imaging Center thoroughly screen all individuals before scans. Claustrophobia and non-compatible implanted devices are listed as exclusion criteria. The confined space and loud noise can be uncomfortable. The study team will prepare the imaging table in advance and provide ear plugs to ensure comfort. *Blood draw:* Routine risks involving laboratory testing include minor pain, bruising, and swelling at the needle site, discomfort of hunger or thirst due to fasting. Less likely is the possibility of lightheadedness, or even briefly feeling faint during the blood drawing procedure.

Genotype. APOE e4 genotype data will be requested from the KU ADC (HSC #11132) for subjects who participate in the KU ADC Clinical Cohort or other studies that share this information back to the Clinical Cohort, and will be generated from all other participants. In some instances, we will also generate the genotype as part of this study. There is potential negative impact if this information were to become known. Thus, this

information will be stored electronically in a secure password protected REDCap database and any paper copies of records will be filed by number in accordance with professional standards of privileged information in a secure badge-access file room at the KU CRC.

Adverse Events: Adverse events are defined as any untoward medical occurrence in study participants or others, which does not necessarily have to be a causal relationship with the study treatment. The seriousness of the adverse event will be determined using the National Cancer Institute Common Terminology Criteria for Adverse Events (CTCAE) v3.0, and any adverse event requiring hospital admission. Adverse events will be assessed only at the visit, but the consent form will have contact information should the participant need to contact the study team regarding delayed development of an AE. Serious Adverse Events will be reported per KUMC requirements.

Safety of the study will be monitored in an ongoing manner by a DSMC according to the DSMC charter and Independent Safety Officer according to a Safety Plan.

Adequacy of Protection Against Risk

a. *Recruitment and Informed Consent:* Recruitment occurs by referrals from organizations serving large numbers of older adults, public service announcements through media, physician referral, other KUMC recruitment sources, and word of mouth. We will also recruit from our pre-existing ADC cohort of participants who have previously given consent to be contacted for future studies. We are very sensitive to issues related to the participant's ability to understand the elements of consent and their capacity to make decisions. We employ a "teach-back" method to confirm understanding of the procedures. Initial screening consent will be acquired verbally over the phone. Written informed consent will be obtained by the PI and/or designated staff for the visit procedures, and the purposes of the study, its assessment procedures, and risks and benefits will be explained. The original signed consent form is kept with the participant's confidential file and a copy is given to the participant.

b. Protection Against Risk: All research data are maintained confidentially in password-protected databases. All paper copies are filed by number in accordance with professional standards of privileged information. Confidentiality is strictly safeguarded by HIPAA-compliant standards.

Additionally, the following policies will be adopted to assure the safety of the participants in this study:
1. Strict adherence to institutional regulations for conducting clinical research
2. The involved support staff will be adequately trained to conduct human research and will have completed the necessary institutional web-based training prior to the study
3. We will comply with HIPPA regulation and commit to maintaining the patient confidentiality.
4. Any research presentations or publications from the proposed studies will not disclose any personal identity information.

Potential Benefits of the Proposed Research to the Participants and Others

Others may benefit for the information gained from this research. In particular, research scientists and clinicians may be able to use the knowledge gained from this study because it will increase our understanding of AD etiology and potentially aid in the design of future therapies.

Importance of the Knowledge to be Gained

Society benefits from research advances in aging and the maintenance of cognitive and brain health. This study will enhance our understanding of the role of cellular metabolism in promoting healthy brain aging. Potential benefits outweigh the risks of this research.

1. Colby SL, Ortman JM. Projections for the Size and Composition of the U. S. Population: 2014 to 2060. In: Bureau USC, ed. Washington, DC2014.

2. Aging NIo. *Growing Older in America: the Healthy and Retirement Study.* Bethesda, MD: National Institutes of Health;2007.

3. Jutkowitz E, Kane RL, Gaugler JE, MacLehose RF, Dowd B, Kuntz KM. Societal and Family Lifetime Cost of Dementia: Implications for Policy. *Journal of the American Geriatrics Society.* Oct 2017;65(10):2169-2175. PMCID: 5657516.
4. Alzheimer's A. 2016 Alzheimer's disease facts and figures. *Alzheimer's & dementia : the journal of the Alzheimer's Association.* Apr 2016;12(4):459-509. PMCID:
5. Yaffe K, Barnes D, Nevitt M, Lui LY, Covinsky K. A Prospective Study of Physical Activity and Cognitive Decline in Elderly Women: Women Who Walk. *Archives of Internal Medicine.* 2001;161(14):1703-1708. PMCID:
6. Weuve J, Kang JH, Manson JE, Breteler MMB, Ware JH, Grodstein F. Physical Activity, Including Walking, and Cognitive Function in Older Women. *JAMA: The Journal of the American Medical Association.* 2004;292(12):1454-1461. PMCID:
7. Nagamatsu LS, Handy TC, Hsu CL, Voss M, Liu-Ambrose T. Resistance training promotes cognitive and functional brain plasticity in seniors with probable mild cognitive impairment. *Arch Intern Med.* Apr 23 2012;172(8):666-668. PMCID: 3514552.
8. Ngandu T, Lehtisalo J, Solomon A, et al. A 2 year multidomain intervention of diet, exercise, cognitive training, and vascular risk monitoring versus control to prevent cognitive decline in at-risk elderly people (FINGER): a randomised controlled trial. *Lancet.* Jun 6 2015;385(9984):2255-2263. PMCID:
9. Maass A, Duzel S, Brigadski T, et al. Relationships of peripheral IGF-1, VEGF and BDNF levels to exercise-related changes in memory, hippocampal perfusion and volumes in older adults. *NeuroImage.* May 1 2016;131:142-154. PMCID:
10. Erickson KI, Voss MW, Prakash RS, et al. Exercise training increases size of hippocampus and improves memory. *Proceedings of the National Academy of Sciences of the United States of America.* Feb 15 2011;108(7):3017-3022. PMCID: 3041121.
11. Vidoni ED, Johnson DK, Morris JK, et al. Dose-Response of Aerobic Exercise on Cognition: A Community-Based, Pilot Randomized Controlled Trial. *PloS one.* 2015;10(7):e0131647. PMCID:
12. Rosenberg A, Ngandu T, Rusanen M, et al. Multidomain lifestyle intervention benefits a large elderly population at risk for cognitive decline and dementia regardless of baseline characteristics: The FINGER trial. *Alzheimer's & dementia : the journal of the Alzheimer's Association.* Oct 19 2017. PMCID:
13. Sink KM, Espeland MA, Castro CM, et al. Effect of a 24-Month Physical Activity Intervention vs Health Education on Cognitive Outcomes in Sedentary Older Adults: The LIFE Randomized Trial. *Jama.* Aug 25 2015;314(8):781-790. PMCID: 4698980.
14. Brasure M, Desai P, Davila H, et al. Physical Activity Interventions in Preventing Cognitive Decline and Alzheimer-Type Dementia: A Systematic Review. *Annals of internal medicine.* Dec 19 2017. PMCID:
15. Barreto PS, Demougeot L, Vellas B, Rolland Y. Exercise training for preventing dementia, mild cognitive impairment, and clinically meaningful cognitive decline: a systematic review and meta-analysis. *The journals of gerontology. Series A, Biological sciences and medical sciences.* Dec 5 2017. PMCID:
16. Daviglus ML, Bell CC, Berrettini W, et al. NIH State-of-the-Science Conference Statement: Preventing Alzheimer's Disease and Cognitive Decline. *NIH Consens State Sci Statements.* Apr 28 2010;27(4). PMCID:
17. Blair SN, Kampert JB, Kohl HW, III, et al. Influences of cardiorespiratory fitness and other precursors on cardiovascular disease and all-cause mortality in men and women. *JAMA: The Journal of the American Medical Association.* 1996;276(3):205-210. PMCID:
18. Lakka TA, Laukkanen JA, Rauramaa R, et al. Cardiorespiratory Fitness and the Progression of Carotid Atherosclerosis in Middle-Aged Men. *Annals of internal medicine.* 2001;134(1):12-20. PMCID:
19. Kurl S, Laukkanen JA, Rauramaa R, Lakka TA, Sivenius J, Salonen JT. Cardiorespiratory Fitness and the Risk for Stroke in Men. *Archives of Internal Medicine.* 2003;163(14):1682-1688. PMCID:
20. Balducci S, Zanuso S, Nicolucci A, et al. Effect of an intensive exercise intervention strategy on modifiable cardiovascular risk factors in subjects with type 2 diabetes mellitus: a randomized controlled trial: the Italian Diabetes and Exercise Study (IDES). *Arch Intern Med.* Nov 8 2010;170(20):1794-1803. PMCID:
21. Knowler WC, Barrett-Connor E, Fowler SE, et al. Reduction in the incidence of type 2 diabetes with lifestyle intervention or metformin. *The New England journal of medicine.* Feb 7 2002;346(6):393-403. PMCID: 1370926.

22. Donnelly JE, Hill JO, Jacobsen DJ, et al. Effects of a 16-month randomized controlled exercise trial on body weight and composition in young, overweight men and women: the Midwest Exercise Trial. *Archives of Internal Medicine.* 2003;163(11):1343-1350. PMCID:
23. Heath GW, Wilson RH, Smith J, Leonard BE. Community-based exercise and weight control: diabetes risk reduction and glycemic control in Zuni Indians. *Am J Clin Nutr.* Jun 1991;53(6 Suppl):1642S-1646S. PMCID:
24. Hagberg JM, Montain SJ, Martin WH, 3rd, Ehsani AA. Effect of exercise training in 60- to 69-year-old persons with essential hypertension. *Am J Cardiol.* Aug 1 1989;64(5):348-353. PMCID:
25. Barbour KA, Blumenthal JA. Exercise training and depression in older adults. *Neurobiology of Aging.* 2005;26(1, Supplement 1):119-123. PMCID:
26. Nelson ME, Rejeski WJ, Blair SN, et al. Physical activity and public health in older adults: recommendation from the American College of Sports Medicine and the American Heart Association. *Med Sci Sports Exerc.* Aug 2007;39(8):1435-1445. PMCID:
27. Defina LF, Willis BL, Radford NB, et al. The association between midlife cardiorespiratory fitness levels and later-life dementia: a cohort study. *Annals of internal medicine.* Feb 05 2013;158(3):162-168. PMCID: PMC3926646.
28. Voss MW, Weng TB, Burzynska AZ, et al. Fitness, but not physical activity, is related to functional integrity of brain networks associated with aging. *NeuroImage.* May 01 2016;131:113-125. PMCID: PMC4837101.
29. Muller J, Chan K, Myers JN. Association Between Exercise Capacity and Late Onset of Dementia, Alzheimer Disease, and Cognitive Impairment. *Mayo Clin Proc.* Feb 2017;92(2):211-217. PMCID:
30. Billinger SA, Vidoni ED, Morris JK, Thyfault JP, Burns JM. Exercise Test Performance Reveals Support of the Cardiorespiratory Fitness Hypothesis. *Journal of aging and physical activity.* Oct 5 2016:1-17. PMCID:
31. Burns JM, Cronk BB, Anderson HS, et al. Cardiorespiratory fitness and brain atrophy in early Alzheimer disease. *Neurology.* Jul 15 2008;71(3):210-216. PMCID:
32. van Boxtel MP, Paas FG, Houx PJ, Adam JJ, Teeken JC, Jolles J. Aerobic capacity and cognitive performance in a cross-sectional aging study. *Med Sci Sports Exerc.* 1997;29(10):1357-1365. PMCID:
33. Shay KA, Roth DL. Association Between Aerobic Fitness and Visuospatial Performance in Healthy Older Adults. *Psychology and aging.* 1992;7(1):15-24. PMCID:
34. Vidoni ED, Honea RA, Billinger SA, Swerdlow RH, Burns JM. Cardiorespiratory fitness is associated with atrophy in Alzheimer's and aging over 2 years. *Neurobiol Aging.* Aug 2012;33(8):1624-1632. PMCID: 3156963.
35. Laurin D, Verreault R, Lindsay J, MacPherson K, Rockwood K. Physical activity and risk of cognitive impairment and dementia in elderly persons. *Arch Neurol.* 2001;58(3):498-504. PMCID:
36. Pignatti F, Rozzini R, Trabucchi M, Yaffe K. Physical Activity and Cognitive Decline in Elderly Persons. *Archives of Internal Medicine.* 2002;162(3):361-362. PMCID:
37. Albert MS, Jones K, Savage CR, et al. Predictors of cognitive change in older persons: MacArthur studies of successful aging. *Psychol Aging.* 1995;10(4):578-589. PMCID:
38. Larson EB, Wang L, Bowen JD, et al. Exercise Is Associated with Reduced Risk for Incident Dementia among Persons 65 Years of Age and Older. *Annals of internal medicine.* January 17, 2006 2006;144(2):73-81. PMCID:
39. Dustman RE, Emmerson RY, Ruhling RO, et al. Age and fitness effects on EEG, ERPs, visual sensitivity, and cognition. *Neurobiol.Aging.* 1990;11(3):193-200. PMCID:
40. Kramer AF, Hahn S, Cohen NJ, et al. Ageing, fitness and neurocognitive function. *Nature.* Jul 29 1999;400(6743):418-419. PMCID:
41. Colcombe SJ, Kramer AF, Erickson KI, et al. Cardiovascular fitness, cortical plasticity, and aging. *Proceedings of the National Academy of Sciences.* 2004;101(9):3316-3321. PMCID:
42. Lautenschlager NT, Cox KL, Flicker L, et al. Effect of physical activity on cognitive function in older adults at risk for Alzheimer disease: a randomized trial. *Jama.* Sep 3 2008;300(9):1027-1037. PMCID:
43. Baker LD, Cross DJ, Minoshima S, Belongia D, Watson GS, Craft S. Insulin resistance and Alzheimer-like reductions in regional cerebral glucose metabolism for cognitively normal adults with prediabetes or early type 2 diabetes. *Arch Neurol.* Jan 2011;68(1):51-57. PMCID: 3023149.
44. Braz ID, Fisher JP. The impact of age on cerebral perfusion, oxygenation and metabolism during exercise in humans. *The Journal of physiology.* Aug 15 2016;594(16):4471-4483. PMCID: 4983626.

45. Van Lieshout JJ, Wieling W, Karemaker JM, Secher NH. Syncope, cerebral perfusion, and oxygenation. *J Appl Physiol (1985).* Mar 2003;94(3):833-848. PMCID:

46. Billinger SA, Craig JC, Kwapiszeski SJ, et al. Dynamics of middle cerebral artery blood flow velocity during moderate-intensity exercise. *J Appl Physiol (1985).* May 1 2017;122(5):1125-1133. PMCID: 5451537.

47. Niwa K, Kazama K, Younkin L, Younkin SG, Carlson GA, Iadecola C. Cerebrovascular autoregulation is profoundly impaired in mice overexpressing amyloid precursor protein. *Am J Physiol Heart Circ Physiol.* Jul 2002;283(1):H315-323. PMCID:

48. Niwa K, Younkin L, Ebeling C, et al. Abeta 1-40-related reduction in functional hyperemia in mouse neocortex during somatosensory activation. *Proceedings of the National Academy of Sciences of the United States of America.* Aug 15 2000;97(17):9735-9740. PMCID: PMC16934.

49. Niwa K, Carlson GA, Iadecola C. Exogenous A beta1-40 reproduces cerebrovascular alterations resulting from amyloid precursor protein overexpression in mice. *J Cereb Blood Flow Metab.* Dec 2000;20(12):1659-1668. PMCID:

50. Farkas E, Luiten PG. Cerebral microvascular pathology in aging and Alzheimer's disease. *Prog Neurobiol.* Aug 2001;64(6):575-611. PMCID:

51. Roher AE, Esh C, Kokjohn TA, et al. Circle of willis atherosclerosis is a risk factor for sporadic Alzheimer's disease. *Arterioscler Thromb Vasc Biol.* Nov 1 2003;23(11):2055-2062. PMCID:

52. Brown WR, Thore CR. Review: cerebral microvascular pathology in ageing and neurodegeneration. *Neuropathology and applied neurobiology.* Feb 2011;37(1):56-74. PMCID: 3020267.

53. Zlokovic BV. Neurovascular pathways to neurodegeneration in Alzheimer's disease and other disorders. *Nature reviews. Neuroscience.* Nov 3 2011;12(12):723-738. PMCID: 4036520.

54. Kim SM, Kim MJ, Rhee HY, et al. Regional cerebral perfusion in patients with Alzheimer's disease and mild cognitive impairment: effect of APOE epsilon4 allele. *Neuroradiology.* Jan 2013;55(1):25-34. PMCID:

55. Bangen KJ, Restom K, Liu TT, et al. Assessment of Alzheimer's disease risk with functional magnetic resonance imaging: an arterial spin labeling study. *J Alzheimers Dis.* 2012;31 Suppl 3:S59-74. PMCID: PMC3443702.

56. Tosun D, Schuff N, Jagust W, Weiner MW, Alzheimer''s Disease Neuroimaging I. Discriminative Power of Arterial Spin Labeling Magnetic Resonance Imaging and 18F-Fluorodeoxyglucose Positron Emission Tomography Changes for Amyloid-beta-Positive Subjects in the Alzheimer's Disease Continuum. *Neurodegener Dis.* 2016;16(1-2):87-94. PMCID:

57. Johnson NA, Jahng GH, Weiner MW, et al. Pattern of cerebral hypoperfusion in Alzheimer disease and mild cognitive impairment measured with arterial spin-labeling MR imaging: initial experience. *Radiology.* Mar 2005;234(3):851-859. PMCID: PMC1851934.

58. Xie L, Dolui S, Das SR, et al. A brain stress test: Cerebral perfusion during memory encoding in mild cognitive impairment. *Neuroimage Clin.* 2016;11:388-397. PMCID: PMC4821452.

59. Jefferson AL, Liu D, Gupta DK, et al. Lower cardiac index levels relate to lower cerebral blood flow in older adults. *Neurology.* Dec 5 2017;89(23):2327-2334. PMCID: 5719926.

60. van Laar PJ, van der Graaf Y, Mali WP, van der Grond J, Hendrikse J, Group SS. Effect of cerebrovascular risk factors on regional cerebral blood flow. *Radiology.* Jan 2008;246(1):198-204. PMCID:

61. Khan TA, Shah T, Prieto D, et al. Apolipoprotein E genotype, cardiovascular biomarkers and risk of stroke: systematic review and meta-analysis of 14,015 stroke cases and pooled analysis of primary biomarker data from up to 60,883 individuals. *International journal of epidemiology.* Apr 2013;42(2):475-492. PMCID: 3619955.

62. Alata W, Ye Y, St-Amour I, Vandal M, Calon F. Human apolipoprotein E varepsilon4 expression impairs cerebral vascularization and blood-brain barrier function in mice. *J Cereb Blood Flow Metab.* Jan 2015;35(1):86-94. PMCID: 4296574.

63. Bell RD, Winkler EA, Singh I, et al. Apolipoprotein E controls cerebrovascular integrity via cyclophilin A. *Nature.* May 16 2012;485(7399):512-516. PMCID: 4047116.

64. Sisante JV, Vidoni ED, Kirkendoll K, et al. Blunted cerebrovascular response is associated with elevated beta-amyloid. *J Cereb Blood Flow Metab.* Jan 1 2017:271678X17732449. PMCID:

65. Brugniaux JV, Marley CJ, Hodson DA, New KJ, Bailey DM. Acute exercise stress reveals cerebrovascular benefits associated with moderate gains in cardiorespiratory fitness. *J Cereb Blood Flow Metab.* Dec 2014;34(12):1873-1876. PMCID: 4269737.

66. Tyndall AV, Davenport MH, Wilson BJ, et al. The brain-in-motion study: effect of a 6-month aerobic exercise intervention on cerebrovascular regulation and cognitive function in older adults. *BMC geriatrics.* Feb 28 2013;13:21. PMCID: 3598522.
67. Leeuwis AE, Hooghiemstra AM, Amier R, et al. Design of the ExCersion-VCI study: The effect of aerobic exercise on cerebral perfusion in patients with vascular cognitive impairment. *Alzheimer's & dementia.* Jun 2017;3(2):157-165. PMCID: 5651416.
68. Rogers RL, Meyer JS, Mortel KF. After reaching retirement age physical activity sustains cerebral perfusion and cognition. *Journal of the American Geriatrics Society.* Feb 1990;38(2):123-128. PMCID:
69. Bailey DM, Marley CJ, Brugniaux JV, et al. Elevated aerobic fitness sustained throughout the adult lifespan is associated with improved cerebral hemodynamics. *Stroke.* Nov 2013;44(11):3235-3238. PMCID:
70. Thomas BP, Yezhuvath US, Tseng BY, et al. Life-long aerobic exercise preserved baseline cerebral blood flow but reduced vascular reactivity to CO2. *Journal of magnetic resonance imaging : JMRI.* Nov 2013;38(5):1177-1183. PMCID: 3695025.
71. Xu X, Jerskey BA, Cote DM, et al. Cerebrovascular perfusion among older adults is moderated by strength training and gender. *Neuroscience letters.* Feb 7 2014;560:26-30. PMCID: 3920729.
72. Alfini AJ, Weiss LR, Leitner BP, Smith TJ, Hagberg JM, Smith JC. Hippocampal and Cerebral Blood Flow after Exercise Cessation in Master Athletes. *Frontiers in aging neuroscience.* 2016;8:184. PMCID: 4974847.
73. Anazodo UC, Shoemaker JK, Suskin N, Ssali T, Wang DJ, St Lawrence KS. Impaired Cerebrovascular Function in Coronary Artery Disease Patients and Recovery Following Cardiac Rehabilitation. *Frontiers in aging neuroscience.* 2015;7:224. PMCID: 4700211.
74. Robertson AD, Marzolini S, Middleton LE, Basile VS, Oh PI, MacIntosh BJ. Exercise Training Increases Parietal Lobe Cerebral Blood Flow in Chronic Stroke: An Observational Study. *Frontiers in aging neuroscience.* 2017;9:318. PMCID: 5626868.
75. Maass A, Duzel S, Goerke M, et al. Vascular hippocampal plasticity after aerobic exercise in older adults. *Molecular psychiatry.* May 2015;20(5):585-593. PMCID:
76. Flodin P, Jonasson LS, Riklund K, Nyberg L, Boraxbekk CJ. Does Aerobic Exercise Influence Intrinsic Brain Activity? An Aerobic Exercise Intervention among Healthy Old Adults. *Frontiers in aging neuroscience.* 2017;9:267. PMCID: 5554511.
77. Smith JC, Paulson ES, Cook DB, Verber MD, Tian Q. Detecting changes in human cerebral blood flow after acute exercise using arterial spin labeling: implications for fMRI. *Journal of neuroscience methods.* Aug 30 2010;191(2):258-262. PMCID:
78. Zlatar ZZ, Bischoff-Grethe A, Hays CC, et al. Higher Brain Perfusion May Not Support Memory Functions in Cognitively Normal Carriers of the ApoE epsilon4 Allele Compared to Non-Carriers. *Frontiers in aging neuroscience.* 2016;8:151. PMCID: 4919360.
79. Thambisetty M, Beason-Held L, An Y, Kraut MA, Resnick SM. APOE epsilon4 genotype and longitudinal changes in cerebral blood flow in normal aging. *Arch Neurol.* Jan 2010;67(1):93-98. PMCID: 2856443.
80. Viticchi G, Falsetti L, Vernieri F, et al. Apolipoprotein E genotype and cerebrovascular alterations can influence conversion to dementia in patients with mild cognitive impairment. *J Alzheimers Dis.* 2014;41(2):401-410. PMCID:
81. Tarumi T, Dunsky DI, Khan MA, et al. Dynamic cerebral autoregulation and tissue oxygenation in amnestic mild cognitive impairment. *J Alzheimers Dis.* 2014;41(3):765-778. PMCID:
82. Prigent-Tessier A, Quirie A, Maguin-Gate K, et al. Physical training and hypertension have opposite effects on endothelial brain-derived neurotrophic factor expression. *Cardiovascular research.* Dec 1 2013;100(3):374-382. PMCID:
83. Banoujaafar H, Van Hoecke J, Mossiat CM, Marie C. Brain BDNF levels elevation induced by physical training is reduced after unilateral common carotid artery occlusion in rats. *J Cereb Blood Flow Metab.* Oct 2014;34(10):1681-1687. PMCID: 4269729.
84. Borror A. Brain-derived neurotrophic factor mediates cognitive improvements following acute exercise. *Medical hypotheses.* Sep 2017;106:1-5. PMCID:
85. Pereira AC, Huddleston DE, Brickman AM, et al. An in vivo correlate of exercise-induced neurogenesis in the adult dentate gyrus. *Proceedings of the National Academy of Sciences of the United States of America.* Mar 27 2007;104(13):5638-5643. PMCID: 1838482.

86. Solvsten CAE, de Paoli F, Christensen JH, Nielsen AL. Voluntary Physical Exercise Induces Expression and Epigenetic Remodeling of VegfA in the Rat Hippocampus. *Mol Neurobiol.* Jan 2018;55(1):567-582. PMCID:

87. Antes R, Salomon-Zimri S, Beck SC, et al. VEGF Mediates ApoE4-Induced Neovascularization and Synaptic Pathology in the Choroid and Retina. *Curr Alzheimer Res.* 2015;12(4):323-334. PMCID:

88. Salomon-Zimri S, Glat MJ, Barhum Y, et al. Reversal of ApoE4-Driven Brain Pathology by Vascular Endothelial Growth Factor Treatment. *J Alzheimers Dis.* Jun 30 2016;53(4):1443-1458. PMCID:

89. Sen A, Nelson TJ, Alkon DL. ApoE isoforms differentially regulates cleavage and secretion of BDNF. *Molecular brain.* Jun 1 2017;10(1):19. PMCID: 5452344.

90. Allard JS, Ntekim O, Johnson SP, et al. APOEepsilon4 impacts up-regulation of brain-derived neurotrophic factor after a six-month stretch and aerobic exercise intervention in mild cognitively impaired elderly African Americans: A pilot study. *Experimental gerontology.* Jan 2017;87(Pt A):129-136. PMCID: 5193139.

91. Wang C, Cui Y, Yang J, et al. Combining serum and urine biomarkers in the early diagnosis of mild cognitive impairment that evolves into Alzheimer's disease in patients with the apolipoprotein E 4 genotype. *Biomarkers : biochemical indicators of exposure, response, and susceptibility to chemicals.* Feb 2015;20(1):84-88. PMCID:

92. Liu YH, Jiao SS, Wang YR, et al. Associations Between ApoEepsilon4 Carrier Status and Serum BDNF Levels--New Insights into the Molecular Mechanism of ApoEepsilon4 Actions in Alzheimer's Disease. *Mol Neurobiol.* 2015;51(3):1271-1277. PMCID:

93. Thow ME, Summers MJ, Summers JJ, Saunders NL, Vickers JC. Variations in the APOE allele or BDNF Val66Met polymorphism are not associated with changes in cognitive function following a tertiary education intervention in older adults: the Tasmanian Healthy Brain Project. *Neurobiol Aging.* Jul 2017;55:175-176. PMCID:

94. Nettiksimmons J, Simonsick EM, Harris T, et al. The associations between serum brain-derived neurotrophic factor, potential confounders, and cognitive decline: a longitudinal study. *PloS one.* 2014;9(3):e91339. PMCID: 3966768.

95. Vidoni ED, Van Sciver A, Johnson DK, et al. A community-based approach to trials of aerobic exercise in aging and Alzheimer's disease. *Contemporary clinical trials.* Aug 7 2012;33(6):1105-1116. PMCID: PMC3468654.

96. Billinger SA, Vidoni ED, Honea RA, Burns JM. Cardiorespiratory response to exercise testing in individuals with Alzheimer's disease. *Archives of physical medicine and rehabilitation.* Dec 2011;92(12):2000-2005. PMCID: 3229718.

97. Morris JK, Vidoni ED, Johnson DK, et al. Aerobic exercise for Alzheimer's disease: A randomized controlled pilot trial. *PloS one.* 2017;12(2):e0170547. PMCID:

98. Vidoni ED, Bothwell RJ, Burns JM, Dwyer JR. Novel recruitment models will drive Alzheimer's trial success. *Alzheimer's & dementia : the journal of the Alzheimer's Association.* Jan 2018;14(1):117-119. PMCID:

99. Galvin JE. The Quick Dementia Rating System (Qdrs): A Rapid Dementia Staging Tool. *Alzheimers Dement (Amst).* Jun 1 2015;1(2):249-259. PMCID: PMC4484882.

100. Ferris LT, Williams JS, Shen CL. The effect of acute exercise on serum brain-derived neurotrophic factor levels and cognitive function. *Med Sci Sports Exerc.* Apr 2007;39(4):728-734. PMCID:

101. Zoladz JA, Pilc A, Majerczak J, Grandys M, Zapart-Bukowska J, Duda K. Endurance training increases plasma brain-derived neurotrophic factor concentration in young healthy men. *Journal of physiology and pharmacology : an official journal of the Polish Physiological Society.* Dec 2008;59 Suppl 7:119-132. PMCID:

102. Fisher JP, Hartwich D, Seifert T, et al. Cerebral perfusion, oxygenation and metabolism during exercise in young and elderly individuals. *The Journal of physiology.* Apr 1 2013;591(7):1859-1870. PMCID: 3624856.

103. Seifert T, Brassard P, Wissenberg M, et al. Endurance training enhances BDNF release from the human brain. *American journal of physiology. Regulatory, integrative and comparative physiology.* Feb 2010;298(2):R372-377. PMCID:

104. Hiura M, Nariai T, Ishii K, et al. Changes in cerebral blood flow during steady-state cycling exercise: a study using oxygen-15-labeled water with PET. *J Cereb Blood Flow Metab.* Mar 2014;34(3):389-396. PMCID: 3948124.

105. Rupp T, Jubeau M, Lamalle L, et al. Cerebral volumetric changes induced by prolonged hypoxic exposure and whole-body exercise. *J Cereb Blood Flow Metab.* Nov 2014;34(11):1802-1809. PMCID: 4269757.

106. Burns JM, Donnelly JE, Anderson HS, et al. Cardiorespiratory Fitness and Brain Atrophy in Early Alzheimer's Disease. *Neurology.* 2008;In Press. PMCID:

107. Tanaka H, Monahan KD, Seals DR. Age-predicted maximal heart rate revisited. *Journal of the American College of Cardiology.* Jan 2001;37(1):153-156. PMCID:

108. Barella LA, Etnier JL, Chang YK. The immediate and delayed effects of an acute bout of exercise on cognitive performance of healthy older adults. *Journal of aging and physical activity.* Jan 2010;18(1):87-98. PMCID:

109. Johnson L, Addamo PK, Selva Raj I, et al. An Acute Bout of Exercise Improves the Cognitive Performance of Older Adults. *Journal of aging and physical activity.* Oct 2016;24(4):591-598. PMCID:

110. Hyodo K, Dan I, Suwabe K, et al. Acute moderate exercise enhances compensatory brain activation in older adults. *Neurobiol Aging.* Nov 2012;33(11):2621-2632. PMCID:

111. Gothe NP, Fanning J, Awick E, et al. Executive function processes predict mobility outcomes in older adults. *Journal of the American Geriatrics Society.* Feb 2014;62(2):285-290. PMCID: PMC3927159.

112. Kramer JH, Mungas D, Possin KL, et al. NIH EXAMINER: conceptualization and development of an executive function battery. *J Int Neuropsychol Soc.* Jan 2014;20(1):11-19. PMCID: PMC4474183.

113. Kamijo K, Hayashi Y, Sakai T, Yahiro T, Tanaka K, Nishihira Y. Acute effects of aerobic exercise on cognitive function in older adults. *The journals of gerontology. Series B, Psychological sciences and social sciences.* May 2009;64(3):356-363. PMCID:

114. Davranche K, Hall B, McMorris T. Effect of acute exercise on cognitive control required during an Eriksen flanker task. *Journal of sport & exercise psychology.* Oct 2009;31(5):628-639. PMCID:

115. Kamijo K, Nishihira Y, Higashiura T, Kuroiwa K. The interactive effect of exercise intensity and task difficulty on human cognitive processing. *International journal of psychophysiology : official journal of the International Organization of Psychophysiology.* Aug 2007;65(2):114-121. PMCID:

116. Kao SC, Westfall DR, Soneson J, Gurd B, Hillman CH. Comparison of the acute effects of high-intensity interval training and continuous aerobic walking on inhibitory control. *Psychophysiology.* Sep 2017;54(9):1335-1345. PMCID:

117. Peiffer R, Darby LA, Fullenkamp A, Morgan AL. Effects of Acute Aerobic Exercise on Executive Function in Older Women. *Journal of sports science & medicine.* Sep 2015;14(3):574-583. PMCID: 4541122.

118. Chen KC, Weng CY, Hsiao S, Tsao WL, Koo M. Cognitive decline and slower reaction time in elderly individuals with mild cognitive impairment. *Psychogeriatrics.* Nov 2017;17(6):364-370. PMCID:

119. Johnson DK, Storandt M, Morris JC, Langford ZD, Galvin JE. Cognitive profiles in dementia Alzheimer disease vs healthy brain aging. *Neurology.* Nov 25 2008;71(22):1783-1789. PMCID:

120. Wang Z, Das SR, Xie SX, et al. Arterial spin labeled MRI in prodromal Alzheimer's disease: A multi-site study. *Neuroimage Clin.* 2013;2:630-636. PMCID: 3777751.

121. Harris PA, Taylor R, Thielke R, Payne J, Gonzalez N, Conde JG. Research electronic data capture (REDCap)--a metadata-driven methodology and workflow process for providing translational research informatics support. *J Biomed Inform.* Apr 2009;42(2):377-381. PMCID: 2700030.
