# Supplementary material for: Dementia risk and dynamic response to exercise: A non-randomized clinical trial
Source: PLoS One. 2022 Jul 8;17(7):e0265860. doi: 10.1371/journal.pone.0265860 (PMC9269742; doi:10.1371/journal.pone.0265860)
Supplement: Attachment — Submitted filename: DYNAMIC_R1_R2R.docx [file pone.0265860.s005.docx]

We appreciate the timely and thoughtful review of our manuscript, *Dementia Risk and Dynamic Response to Exercise: A Non-randomized Clinical Trial*, provided by the reviewers. We have carefully considered each critique and provided our response below. To assist reviewers, in most cases we have provided the critique in bold, our response in plain text, and the revision to the manuscript in *“quoted italics”*.

**Reviewer #1
Minor revisions:
1- Abstract: In addition to the count, include the percentage female.**

We have added the percentage female to the abstract

*“Methods: Sixty-two older adults (73±5 years old, 41 female [67%])…”*

**2- Line 193: Clarify if the t-tests for comparing pre- to post-exercise change were paired t-test.**

We apologize for the lack of clarity. We performed independent t-tests to compare APOE4 carrier and non-carrier groups. The measures of interest were 1) the post-exercise area under the curve, and 2) the pre-to-post exercise change [change measure] in neurotrophin concentration. We have revised our language for improved clarity.

*“Our a priori planned analysis of the primary CBF outcome measure was an independent t-test comparison of CBF AUC between APOE4 carriage groups, assuming unequal variance. Our secondary outcomes were tested via independent t-test comparison of change in blood-based neurotrophic marker levels (post-exercise minus pre-exercise concentration) between e4carriage groups.”*

**3- Line 195: State the underlying covariance structure used in the linear mixed effects model and the criteria for selecting it.**

Since we fit a linear mixed effects model with a random intercept, the resulting covariance structure for our observations is compound symmetry. We have stated this in the text at the specified location.

**4- Specify the level of significance. For instance, add the following statement, filling in the value for x.xx. P-values less than x.xx were considered statistically significant.**

Thank you for identifying this oversight. We have clarified our a priori defined alpha.

*“For all analyses we set α = 0.05.”*

**5- Use consistent notation for standard deviation. The standard notation is SD. Spell out the abbreviation at its first use.**

We have corrected the use of “SD” to “standard deviation”. No abbreviation is necessary in the manuscript as it is only used twice in the footnotes of tables.

**Reviewer #2**

**1. Choose a more descriptive short title.**

We have revised the Short Title.

*“Short Title: Cerebral blood flow response to exercise”*

**2. Apolipoprotein E, first illustrate the abbreviation then mention it.**

This has been corrected.

**3. The results of previous study should not be mentioned in abstract section it should be in the discussion**

Reference to prior studies have been removed from the abstract Conclusions section

*“Results from this study provide an initial characterization of the acute brain blood flow and neurotrophin responses to a bout of exercise in older adults with and without this known risk allele for cardiovascular disease and Alzheimer’s disease.”*

**4. Where is the key words**

Keywords were entered into the PLOS One submission management system. We have added them to the manuscript.

*“Keywords: cerebral blood flow; aerobic exercise; Alzheimer's disease; perfusion”*

**5.** **Explain the rationale of the study. Please delete information unrelated to objective so that the section is short and sweet. Kindly focus on three elements of introduction.
a. What is known about the topic? (Background)
b. What is not known? (The research problem)
c. Why the study was done? (Justification)
Objective is not clear as mentioned above.**

Thank you for the opportunity to clarify the rationale and objective of the present study. We have edited the Introduction section to improve the clarity and more concisely motivate our primary objectives. Of particular note, we have clarified our statement on the scientific knowledge gap and study premise.

*“… whether older individuals who possess APOE4 show differential CBF responses to an acute bout of exercise has not been investigated. Understanding acute physiologic responses to is important as any benefits of exercise will necessarily result from the cumulative effects of these brief regular exercise exposures.*

*To further characterize potential intermediary mechanisms between exercise and brain health we designed the present study to assess the immediate CBF response to a single bout of acute exercise.”*

**6. Why did you choose MRI explain ? and add references to all measures**

Thank you for prompting this addition. In addition to our detailed description and appropriate references for the PCASL analyses procedure, we have added the following statement and reference.

“*Arterial spin labeling via magnetic resonance imaging was chosen to capture CBF due to its advantage in spatial localization and ability to yield a physiologically quantifiable outcome.(Borgovac et al, 2012)”*

Since the time of original submission, we have come across new methodologies for image processing that may improve the quality of signal processing. To improve the rigor of this work, we have implemented these new analyses approaches in this manuscript revision, including the use of smoothing of the PCASL signal, and have updated citations, and CBF measures reported in the results and Table 2. The outcomes of our statistical analyses remained similar after implementation of the revised CBF analyses methodology and our interpretation of the findings is unchanged.

**7. Mention who did blood specimen processing**

We have added reference to who performed the blood specimen processing.

*“Blood specimen processing was performed by a trained phlebotomist. Subsequent analyses were performed by trained staff, overseen by an experienced co-investigator (JKM).”*

**8. Results need to follow ABC (accuracy, brevity, clarity)
Kindly frame it along the following elements of results
i. Text to tell the story
ii. Tables to summarize the evidence
iii. Figures to highlight the main findings**

In compliance with the study sponsors at the National Institutes of Health (NIH), we have followed standard NIH Consolidated Standards of Reporting of Trials (CONSORT) guidelines for reporting clinical trial results: <http://www.consort-statement.org/> .

. We have specified primary and secondary outcomes, provided tables to summarize the main evidence, andfigures to highlight the main findings.

**9.This part [enrollment] should be placed in the method section not the results**

We appreciate the perspective on the optimal location for the enrollment narrative. Throughout the manuscript we have followed standard CONSORT reporting. CONSORT defines the enrollment narrative as part of the Results (Items 13a/b and 14a/b on the CONSORT checklist).

**10. Why didn’t you do intention to treat analysis for this drop out**

We appreciate this concern, and after further consideration we feel that as only 3 people were missing for the primary outcome the number of remaining subjects included in our analysis is sufficient for estimation and statistical inference. Thus, we opted for this approach as opposed to risk inducing potential biases through imputation.

**11. State the underlying covariance structure used in the linear mixed effects model and the criteria for selecting it.**

Since we fit a linear mixed effects model with a random intercept, the resulting covariance structure for our observations is compound symmetry.

We have stated this in the methods section:

“*We also tested an exploratory linear mixed effects model with a random intercept coefficient for each participant and a covariance structure of compound symmetry*.”

**12. Describe sources of potential bias and imprecision.**

We agree that further elaboration on sources of potential bias and imprecision would improve the readers’ ability to carefully evaluate the results of the present study. We have edited the Discussion section to include the following limitations:

“…*Given the advantage of high spatial resolution and sensitivity to cerebral perfusion changes, the present study utilized a MR imaging method to quantify cerebral blood flow. This method limits our ability to interpret CBF changes during the exercise bout that may have influenced immediate post-exercise CBF changes. Though this MR imaging method is currently regarded as the most accurate and precise method to quantify cerebral blood flow, the CBF measurements may be sensitive to factors such as day-to-day variability and circadian cycle (Heijtel et al 2014).”*

“…*Given the strong link between cardiovascular health and cognition with aging, our exclusion of older adults with cognitive impairment or dementia could have biased our sample towards individuals with higher vascular health than the typical older adult population.*”

**13. Generalizability of the trial findings need to be put.**

We have included the following statement in the Limitations.

*“Finally, though our sample demonstrates racial and socioeconomic diversity, both inclusion criteria that limited the enrollment of individuals with severe cardiovascular disease and the underrepresentation of other racial identities, men, and additional sources of diversity limit the broad generalizability of this work.”*
