# Supplementary material for: Dementia risk and dynamic response to exercise: A non-randomized clinical trial
Source: PLoS One. 2022 Jul 8;17(7):e0265860. doi: 10.1371/journal.pone.0265860 (PMC9269742; doi:10.1371/journal.pone.0265860)
Supplement: Attachment — Submitted filename: DYNAMIC_R2_R2R.docx [file pone.0265860.s006.docx]

We appreciate the timely review of our first revision of our manuscript, *Dementia Risk and Dynamic Response to Exercise: A Non-randomized Clinical Trial*, provided by the reviewers. We have performed the final requested changes.

**Reviewer #1: Minor revision:**
The following statement is vague since the denotation of the lower case a is nonstandard. "For all analyses we set a= 0.05." Consider writing, "P-values less than 0.05 were considered statistically significant."

We have changed the statement in the manuscript as requested.
